# Identification of a basement membrane-related gene signature for predicting prognosis, immune infiltration, and drug sensitivity in colorectal cancer

**DOI:** 10.3389/fonc.2024.1428176

**Published:** 2024-07-01

**Authors:** Xiang Shengxiao, Sun Xinxin, Zhu Yunxiang, Tang Zhijie, Tang Xiaofei

**Affiliations:** ^1^ Department of Science and Education, Suqian First Hospital, Suqian, Jiangsu, China; ^2^ Department of Science and Education, Yangzhou Maternal and Child Health Hospital, Yangzhou, Jiangsu, China; ^3^ Department of Gastrointestinal Surgery, The Affiliated Hospital of Yangzhou University, Yangzhou University, Yangzhou, China

**Keywords:** basement membrane, colorectal cancer, prognostic signature, nomogram, immunity, tumor microenvironment

## Abstract

**Background:**

Colorectal cancer (CRC) is the most common malignancy affecting the gastrointestinal tract. Extensive research indicates that basement membranes (BMs) may play a crucial role in the initiation and progression of the disease.

**Methods:**

Data on the RNA expression patterns and clinicopathological information of patients with CRC were sourced from The Cancer Genome Atlas (TCGA) and the Gene Expression Omnibus (GEO) databases. A BM-linked risk signature for the prediction of overall survival (OS) was formulated using univariate Cox regression and combined machine learning techniques. Survival outcomes, functional pathways, the tumor microenvironment (TME), and responses to both immunotherapy and chemotherapy within varying risk classifications were also investigated. The expression trends of the model genes were evaluated by reverse transcription polymerase chain reaction (RT-PCR) and the Human Protein Atlas (HPA) database.

**Results:**

A nine-gene risk signature containing UNC5C, TINAG, TIMP1, SPOCK3, MMP1, AGRN, UNC5A, ADAMTS4, and ITGA7 was constructed for the prediction of outcomes in patients with CRC. The expression profiles of these candidate genes were verified using RT-PCR and the HPA database and were found to be consistent with the findings on differential gene expression in the TCGA dataset. The validity of the signature was confirmed using the GEO cohort. The patients were stratified into different risk groups according to differences in clinicopathological characteristics, TME features, enrichment functions, and drug sensitivities. Lastly, the prognostic nomogram model based on the risk score was found to be effective in identifying high-risk patients and predicting OS.

**Conclusion:**

A basement membrane-related risk signature was constructed and found to be effective for predicting the prognosis of patients with CRC.

## Introduction

Colorectal cancer (CRC) is a prevalent malignancy affecting the gastrointestinal tract. In 2023, approximately 153 000 individuals in the USA were diagnosed with CRC, with nearly 52 000 succumbing to the disease (1). Over time, a spectrum of therapeutic modalities for CRC, encompassing surgery, radiation, and chemotherapy, have significantly improved patient prognoses (2). Nonetheless, these interventions do not always yield favorable results. Certain individuals experience suboptimal outcomes attributable to cancer recurrence, metastasis, or drug resistance (3–5). Molecular alterations, both genetic and non-genetic, contribute substantially to both the onset and progression of CRC. Hence, it is imperative to pinpoint novel biomarkers for improving treatment efficacy and develop predictive models to steer the management of patients with CRC.

The basement membrane (BM) represents a distinctive form of extracellular matrix (ECM) that surrounds cells. The BM is composed predominantly of collagen IV, laminin, proteoglycans, nidogens, and growth factors (6, 7). A fully intact BM is essential for preventing the infiltration of tumor cells into the surrounding stromal tissue, and thus acts as a critical physical barrier to tumor cell metastasis (7, 8). Dysregulation of the BM can increase tumor cell migration and invasiveness (9). Moreover, several studies have reported that the BM can control the differentiation, cell polarity, and survival of tumor cells (10, 11). A study by Jayadev and his team observed a network of 222 BM-related genes (BMGs), highlighting the complex nature of the BM and how it can affect human health (12). Certain features of the BM have been demonstrated to impact the prognosis of patients with liver, stomach, and breast cancer (13–15). Despite this, no studies have investigated whether these BMGs could affect the survival rates of patients with CRC.

In this study, we constructed a predictive model that incorporated nine BMGs to estimate survival outcomes for patients with CRC. This model also sheds light on the status of the tumor microenvironment (TME) and predicts how the patient might respond to chemotherapy. The findings provide novel insights for prognostic evaluation and decision-making in the management of patients with CRC. Moreover, they open new avenues for exploring more effective treatment strategies for patients with CRC.

## Materials and methods

### Study design

**Figure 1:**
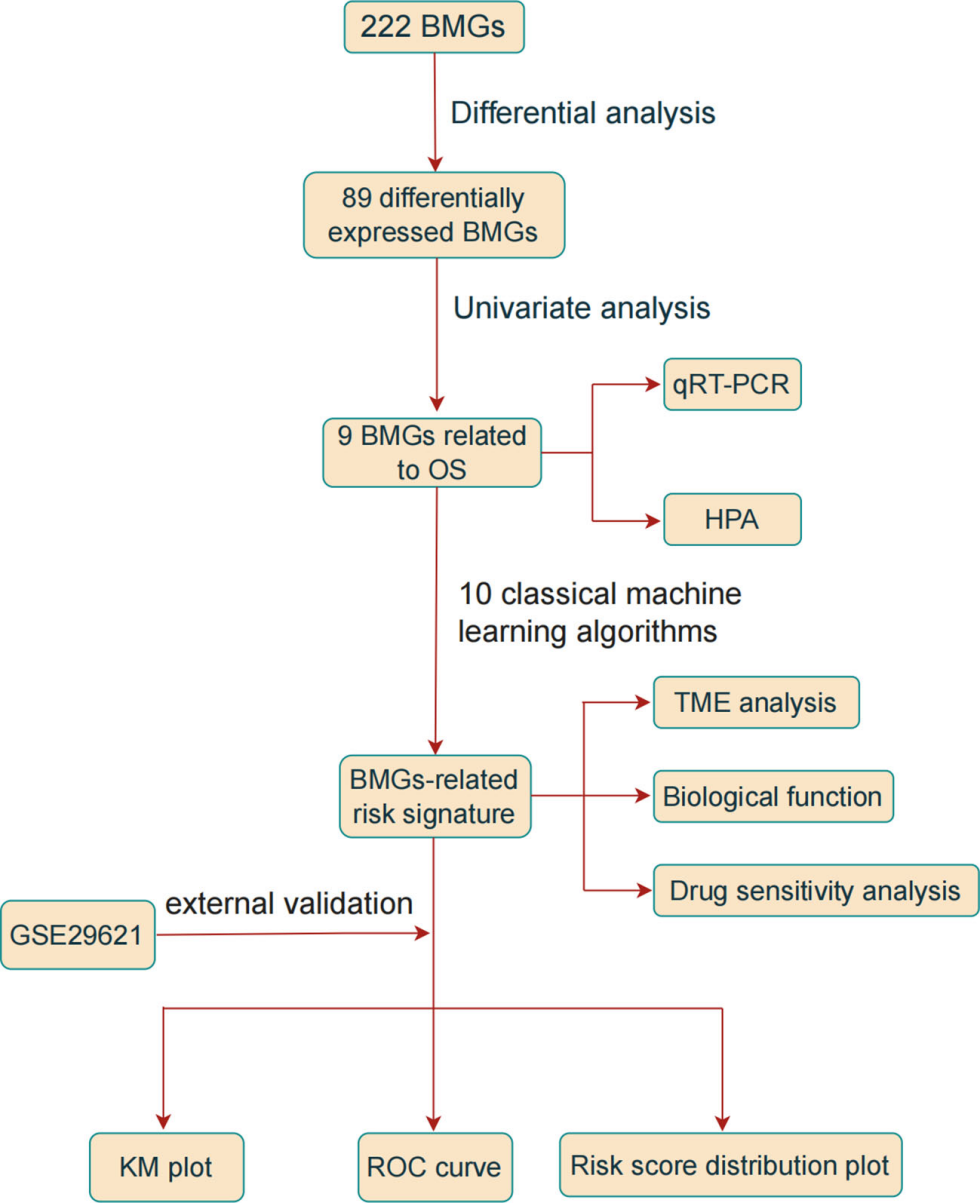
Study Flow Diagram.

**Figure 1** illustrates a flowchart of the study, providing a comprehensive overview of the entire study process.

### Data collection and preparation

Data on the RNA expression patterns and clinicopathological information of 624 CRC tissues and 51 normal colonic tissues were acquired from The Cancer Genome Atlas (TCGA) database. Additional data were obtained from the GSE29621 dataset from the Gene Expression Omnibus (GEO) database; these data were used as the validation set (16, 17). The TCGA-CRC dataset was incorporated with the GEO data and the “ComBat” algorithm from the “sva” R package was used for the correction of batch effects.

### Identification of differentially expressed genes

A set of 224 BMGs was obtained from a previous study for subsequent analysis (12). Differentially expressed BMGs (DEBMGs) between normal and tumor samples were identified using the R package “limma”. Using the criteria of an absolute log2 fold-change (FC) above 1 and a false discovery rate (FDR) below 0.05, a total of 89 DEBMGs were identified.

### Establishment of a prognostic model for BM

To investigate the associations between the identified DEBMGs and the overall survival (OS) rates of patients with CRC, a univariate Cox regression analysis was conducted to identify prognostic markers, using the criterion of a p-value less than 0.05, signifying statistical significance.

A comprehensive analytical process was then followed, integrating 10 classical machine-learning algorithms and 117 algorithm combinations to devise an accurate and robust prognostic signature derived from characteristics of the BM (18). These algorithms included supervised principal components (SuperPC), random forest (RF), CoxBoost, least absolute shrinkage and selection operator (LASSO), survival support vector machine (Survival-SVM), partial least squares regression for Cox (plsRcox), gradient boosting machine (GBM), ridge regression, elastic network (Enet), and Stepwise Cox. The efficacy of each model was assessed using the concordance index (C-index) for both the training and validation sets, and the model with the top average C-index was considered the most suitable.

### Analysis of the tumor microenvironment and gene set variation analysis

The “ESTIMATE” package in R was used to analyze the TME in terms of different risk categories (19). In parallel, immune cell infiltration was assessed using single-sample gene set enrichment analysis (ssGSEA) from the “gsva” package in R. Furthermore, 50 hallmark gene sets were identified from the MsigDB database and the gene set variation analysis (GSVA) algorithm was used for the comprehensive evaluation of each gene set, thereby assessing potential changes in the biological functions of the different risk groups (20).

### Therapy response prediction

The TIDE computational method was employed to analyze the likelihood of patients with CRC responding to immunotherapy. A higher TIDE score indicates an increased chance of evading immune response during such therapy (21). Furthermore, the “Oncopredict” package was used to predict the response of patients with CRC to commonly prescribed medications (22).

### Exploration of expression patterns

Human intestinal epithelial cells (FHCs) and the human colorectal cancer cell line HCT8 were procured from Punosai Life Science and Technology Co., Ltd. The cells were cultured in DMEM, supplemented with 10% fetal bovine serum, and maintained at 37°C under a 5% CO_2_ environment. RNA isolation was performed using TRIzol reagent, followed by reverse transcription with PrimeScript™ RT Master Mix. The housekeeping gene, GAPDH, was used as an internal control. The sequences of the primers are presented in **Table 1**. Immunohistochemical information from the HPA database was used to investigate the protein expression of the genes.

**Table 1:** Primers used for RT-PCR.

Gene symbol	Primer sequence (5′ to 3′)
ITGA7	Forward: TGCTGGTGCTGCTCCTGTG
	Reverse: TCTTCTCCTCCTTGAACTGCTGTC
ADAMTS4	Forward: CTGACTTCCTGGACAATGGCTATG
	Reverse: GCTGTGGACAATGGCGTGAG
UNC5A	Forward: GCCGTCTGCCTGGTCCTG
	Reverse: TGGAAGCCTGAGGTGAGAATGG
AGRN	Forward: GATGGAGTCACATACGGCAACG
	Reverse: TCACAGTCACGGAGGCAGATG
MMP1	Forward: TTACACGCCAGATTTGCCAAGAG
	Reverse: TTACACGCCAGATTTGCCAAGAG
SPOCK3	Forward: AATAATGAGTGGTGCTACTGCTTCC
	Reverse: TGCCGCTTCTGAATATTGCTGAG
TIMP1	Forward: CCTGTTGTTGCTGTGGCTGATAG
	Reverse: ACGCTGGTATAAGGTGGTCTGG
UNC5C	Forward: CGGACTGGGACTGGGATACTTG
	Reverse: GAGGCTCAGGTGGATCAGAAGG
TINAG	Forward: TATGCGGCGAATGCGTTGTG
	Reverse: TACCAAGGCTGTGTGTGAGGAG
GAPDH	Forward: GGAGCGAGATCCCTCCAAAAT
	Reverse: GGCTGTTGTCATACTTCTCATGG

### Statistical analysis

R software (version 4.1.1) was used for all data analysis and for generating visual illustrations. Two-sided P-values <0.05 were considered statistically significant.

## Results

### Identification of DEBMGs and development of the prognostic signature

**Figure 2:**
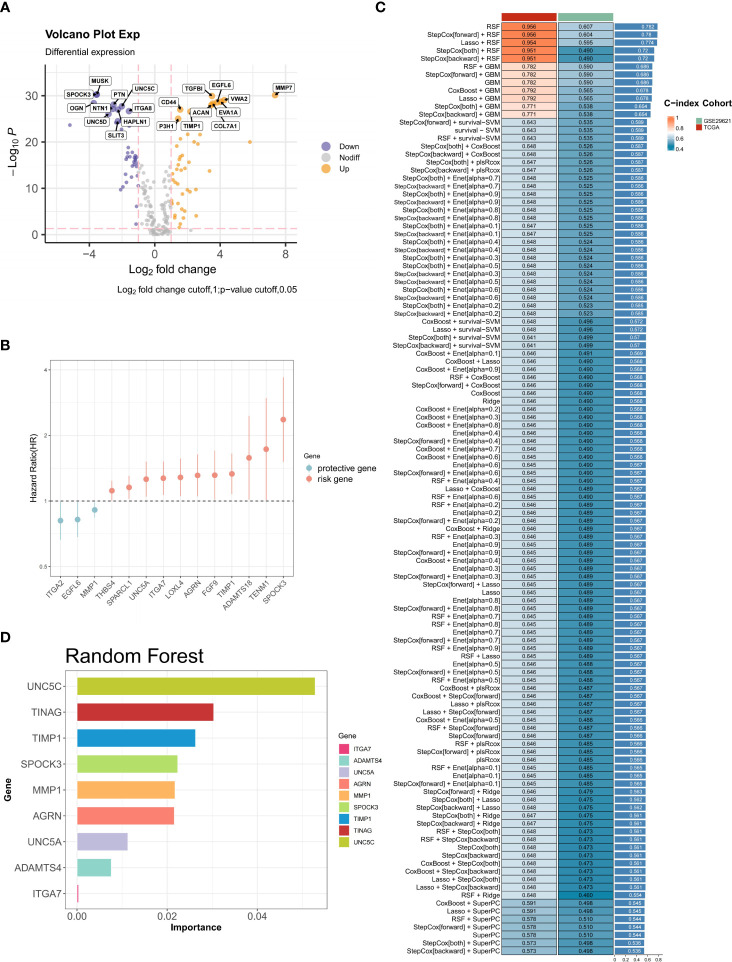
Construction of BM-related risk features. **(A)** Volcano plot of the levels of differentially expressed BMGs. **(B)** Univariate Cox regression analysis. **(C, D)** RF was chosen as the best model with the highest average C-index.

In the initial assessment, the expression patterns of 224 BMGs in both tumor and normal samples were examined in the TCGA-CRC cohort. Analysis of differential expression led to the identification of 89 DEBMGs, 46 of which were upregulated and 42 downregulated (**Figure 2A**). Subsequently, nine DEBMGs that were shown by univariate Cox regression to be significantly associated with OS were identified (**Figure 2B**). After the implementation of 117 algorithm combinations for the development of the prediction model, the mean C-index was calculated for each algorithm in the TCGA-CRC and GSE29621 datasets. As depicted in **Figures 2C, D**, RF was selected as the best model due to its superior average C-index of 0.782.

### Evaluation of the performance of the BMG-related risk signature

**Figure 3:**
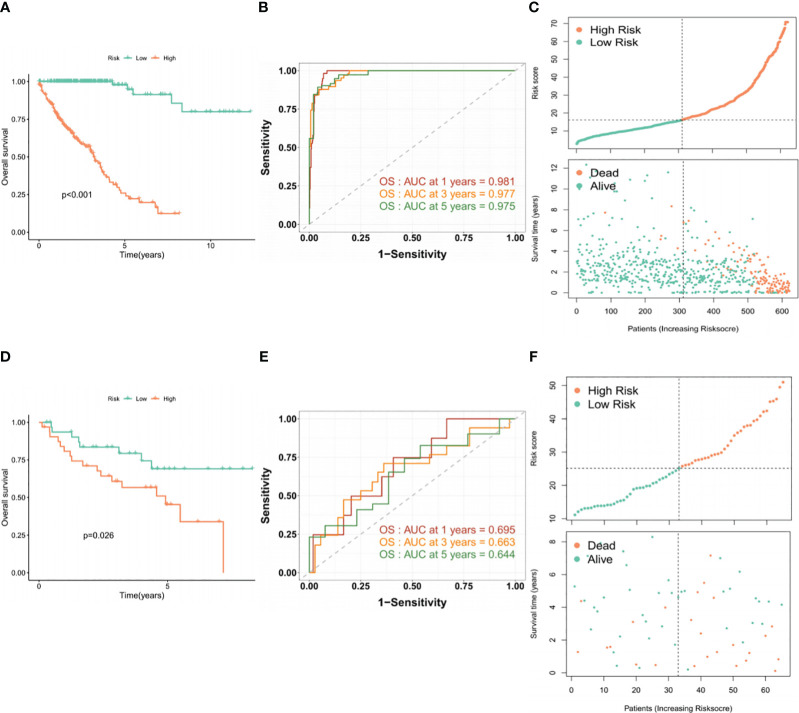
Evaluation of the performance of the BMG-related risk signature. **(A-C)** KM, ROC, and risk distribution plots in the TCGA-CRC dataset. **(D-F)** KM, ROC, and risk distribution plots in the GSE29621 dataset.

To assess the prognostic performance of the BMG-related model, patients were stratified into high- and low-risk categories using the median risk score as the cutoff. The Kaplan-Meier (KM) curve demonstrated that the high-risk group displayed markedly worse survival outcomes (**Figure 3A**). The areas under the receiver operating characteristic (ROC) curve (AUCs) for 1-, 3-, and 5-year OS were 0.981, 0.977, and 0.975, respectively (**Figure 3B**). In addition, the visual representation of risk score distribution clearly showed that patients classified as high-risk had significantly higher mortality rates (**Figure 3C**). These findings were confirmed in the validation set, underscoring the consistent and robust performance of the BMG-related risk signature (**Figure 3D–F**).

**Figure 4:**
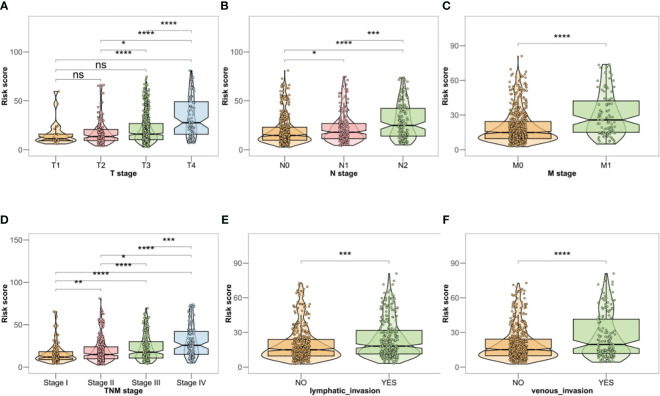
**(A–F)** Clinical correlation analysis in TCGA-CRC database. *P < 0.05, **P < 0.01, ***P < 0.001, ****P < 0.0001, ns, no significant.

Investigation of the association between the risk profile and clinical data indicated significant differences in the TNM stage, venous infiltration, and lymphatic penetration between the high- and low-risk groups. These findings suggest that the risk profile related to BMGs could potentially serve as a predictive marker for clinical traits in patients with CRC (**Figures 4A–F**).

### The TME and functional analysis

**Figure 5:**
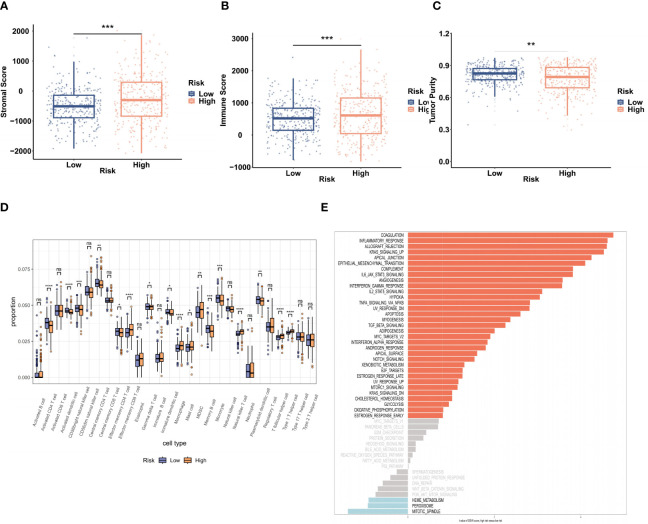
Evaluation of the TME and biological functions. **(A–C)** The stromal score, immune score, and tumor purity in the two risk groups were evaluated by ESTIMATE analysis. **(D)** ssGSEA analysis of the infiltration of 28 immune cell types. **(E)** GSVA of 50 hallmark pathway enrichments. **P < 0.01, ***P < 0.001.

The ESTIMATE algorithm was used to examine the relationship between the TME and the BMG-related risk signature. Notably, patients in the high-risk group showed significantly elevated stromal and immune scores, along with decreased tumor purity, in contrast to patients categorized as low-risk (**Figures 5A–C**). This implies that patients with an increased BMG-related risk score had a more active and resilient immune response. Furthermore, the ssGSEA algorithm provided additional evidence by confirming substantial disparities in the immune cell infiltration levels between the two patient cohorts (**Figure 5D**). Specifically, the high-risk TCGA-CRC group showed significant increases in the infiltration of macrophages, mast cells, and type 1 T helper cells, together with marked reductions in the proportions of CD4+ T cells, activated dendritic cells, and CD56 bright natural killer cells. Lastly, the GSVA method to investigate alterations in hallmark pathway enrichments between the two risk groups (**Figure 5E**). Notably, 33 tumor-associated hallmark pathways (66%) exhibited significant enrichment in the high-risk groups, indicating a higher likelihood of tumor cell invasion in these patients.

### Drug sensitivity analysis

**Figure 6:**
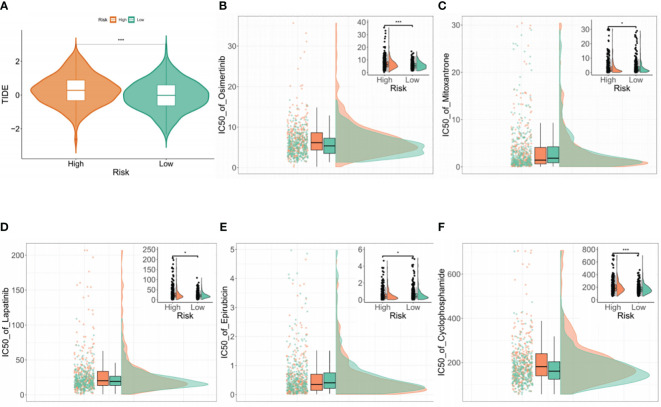
Drug sensitivity analysis **(A)** Comparison of TIDE scores between the high- and low-risk groups. **(B–F)** Drug sensitivity analysis of the two groups using the “oncoPredict” tool.

The TIDE algorithm was used to assess the ability of the BMG-related risk score to predict patient response to cancer immunotherapy. This showed significantly elevated TIDE scores in the high-risk group, indicating a potentially higher likelihood of positive responses to immunotherapy (**Figure 6A**). To confirm the association between the BMG-related risk signature and its clinical application, the “oncoPredict” tool was used to assess the sensitivities to commonly used chemotherapy drugs. A decreased IC50 value implies heightened drug sensitivity and a more favorable therapeutic outcome. The findings indicated that the low-risk group was predicted to be more sensitive to ocittinib, lapatinib, and cyclophosphamide, while the high-risk group showed greater sensitivity to mitoxantrone and epirubicin (**Figures 6B–F**).

### RT-PCR and HPA

**Figure 7:**
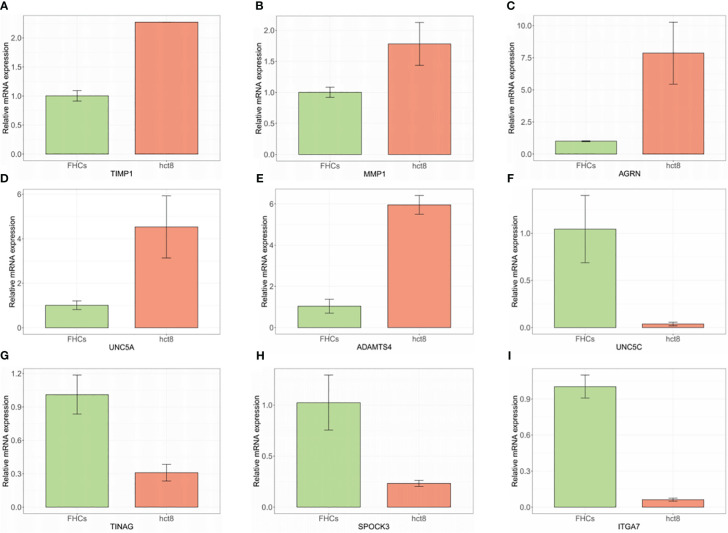
**(A–I)** mRNA levels of BMGs in HCT8 CRC and normal FHC cells measured by RT-PCR.

The expression of 9 BMGs was verified in the FHC human intestinal epithelial cell and HCT8 human colorectal cancer cell lines using RT-PCR. Increased expression of TIMP1, MMP1, AGRN, UNC5A, and ADAMTS4 was observed, while the expression of UNC5C, TINAG, SPOCK3, and ITGA7 was reduced in the normal FHC cells compared to the HCT8 tumor cells (**Figure 7**).

**Figure 8:**
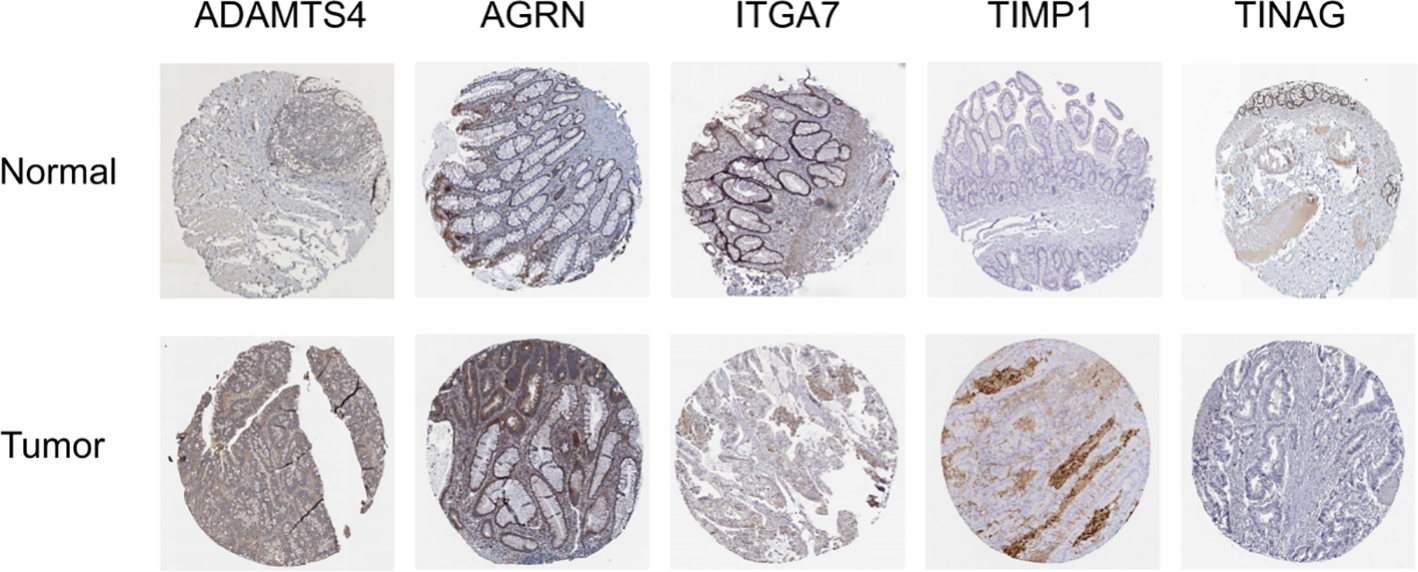
Immunohistochemical results from the HPA database.

Additionally, protein expression of the BMGs was explored using the HPA tool. The protein expression of ADAMTS4, AGRN, ITGA7, TIMP1, and TINAG was consistent with the PCR results. Unfortunately, the HPA database did not provide any data on the expression of MMP1, SPOCK3, UNC5A, and UNC5C (**Figure 8**).

### Nomogram construction and evaluation

**Figure 9:**
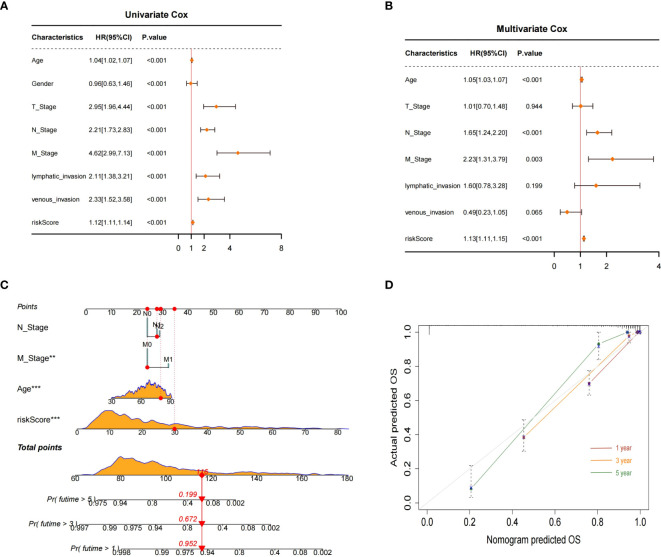
Nomogram construction and evaluation. **(A, B)** Univariate and multivariate Cox analyses. **(C)** Nomogram for predicting 1-, 3-, and 5-year OS probabilities for patients with CRC. **(D)** Calibration plot of the nomogram.

Both univariate and multivariable Cox regression analyses were performed to identify independent predictors of prognosis for patients with CRC. The results of the univariate Cox analysis indicated that age, TNM stage, lymphatic invasion, venous invasion, and the BMG-related risk signature were significantly associated with CRC prognosis (**Figure 9A**). Subsequently, the multivariable Cox regression analysis identified age, stages N and M, and the risk signature as independent prognostic factors for CRC patients (**Figure 9B**). These findings were used to construct a nomogram to predict the likelihood of OS for CRC patients at the 1-, 3-, and 5-year time points (**Figure 9C**). The calibration curve showed a high level of agreement between the predicted survival rates from the nomogram and actual survival rates (**Figure 9D**).

## Discussion

CRC is the most common malignancy of the gastrointestinal tract. The occurrence of distant metastasis is a critical determinant of the disease prognosis (23). Extensive evidence has confirmed that tumor metastasis is frequently associated with the degradation or disruption of the BM (7, 8). Moreover, the BM is involved in the regulation of various cell functions, such as cell polarity, differentiation, and survival (10, 11). The development of risk scores with specific attributes to evaluate the prognosis of CRC patients from various angles has been demonstrated as a feasible approach (24). However, to date, there is a lack of CRC prognostic models that incorporate the BM. The present study identified nine differentially expressed BMGs that were linked to CRC prognosis. Integration of 10 machine learning algorithms led to the development of a novel prognostic model based on the BMGs, which was found to be effective for predicting the prognosis of patients with CRC. Furthermore, the risk signature could accurately predict both clinicopathological features associated with prognosis and the sensitivity of patients to drugs commonly used for treating CRC.

Previous research has shown that all these BMGs play crucial roles in tumorigenesis and tumor progression. TINAG, an extracellular matrix protein expressed in the BM, has recently been found by Zhang et al. to be involved in the migration, proliferation, and invasion of hepatocellular carcinoma (25). UNC5C, identified as downregulated in CRC, acts as a tumor suppressor and is linked to tumor progression in colorectal malignancies (26, 27). MMP1 is known to be overexpressed in various cancer types and associated with both tumor development and metastasis and, intriguingly, surfaced as a defensive element against CRC (28–30). ADAMTS4, a member of the ADAMTS family, has been shown to promote tumorigenesis in glioblastomas, melanoma, prostate cancer, and other cancers (31–33), while ITGA7, another ECM-binding protein, was found by Liu et al. to inhibit CRC cell growth and metastasis (34) and UNC5A serves as a transmembrane receptor, mediating ligand-dependent signaling pathways that regulate cell survival or induce cell death (35).

Originally recognized as a facilitator of N-type acetylcholine receptors at the neuromuscular junction, AGRN is expressed in the cell membrane where it is involved in controlling neuromuscular communication (36). Zhang et al. discovered that AGRN promotes the progression of lung cancer through the Notch signaling pathway activation, suggesting its potential as a therapeutic target (37). TIMP1 has been found to regulate various tumorigenic processes, including proliferation, apoptosis, and metastasis (38). In Song’s research, a strong association between TIMP1 overexpression and CRC recurrence and aggressiveness was demonstrated, highlighting its potential as a viable biomarker for prognosis prediction in CRC (39). SPOCK3, a proteoglycan, is secreted into the extracellular matrix, and Luo et al. reported that SPOCK3 was associated with prostate cancer progression by controlling the infiltration of immune cells (40).

Our research has several limitations that should be acknowledged. Firstly, the study population comprised CRC samples obtained from publicly available databases. Hence, it is important to verify the precision of the BMG risk signature in practical CRC cohorts. Furthermore, despite verification of the expression of BMGs through PCR and immunohistochemistry, the precise mechanisms underlying the functions of these genes in CRC are still not fully understood, requiring additional clarification through *in vivo* and *in vitro* studies.

In summary, a novel BM-related scoring system was constructed that could effectively predict the prognosis of patients with CRC. This scoring system has potential as a valuable tool for guiding clinical treatment decisions, providing clinicians with a reliable reference for personalized treatment strategies. These findings have the potential to significantly advance the development of tailored therapeutic approaches for CRC.

## Data availability statement

The original contributions presented in the study are included in the article/supplementary material. Further inquiries can be directed to the corresponding author.

## Ethics statement

Ethical approval was not required for the studies on humans in accordance with the local legislation and institutional requirements because only commercially available established cell lines were used. Since all the data used in the current study was available online, and no individual patient was involved, the IRB (Institutional Review Board) review was exempted.

## Author contributions

XS: Data curation, Formal analysis, Funding acquisition, Investigation, Methodology, Project administration, Software, Writing – original draft. SX: Conceptualization, Data curation, Formal analysis, Project administration, Resources, Software, Supervision, Validation, Visualization, Writing – original draft. ZY: Conceptualization, Investigation, Methodology, Project administration, Resources, Visualization, Writing – review & editing. TZ: Formal analysis, Investigation, Methodology, Project administration, Resources, Writing – review & editing. TX: Data curation, Funding acquisition, Investigation, Methodology, Resources, Validation, Writing – review & editing.
